# Sex differences exist in adult heart group 2 innate lymphoid cells

**DOI:** 10.1186/s12865-022-00525-0

**Published:** 2022-10-31

**Authors:** Hongyan Peng, Shuting Wu, Shanshan Wang, Qinglan Yang, Lili Wang, Shuju Zhang, Minghui Huang, Yana Li, Peiwen Xiong, Zhaohui Zhang, Yue Cai, Liping Li, Youcai Deng, Yafei Deng

**Affiliations:** 1 Pediatrics Research Institute of Hunan Province, Hunan Children’s Hospital, Changsha, 410007 China; 2 Hunan Provincial Key Laboratory of Children’s Emergency Medicine, Hunan Children’s Hospital, Changsha, 410007 China; 3 State Key Laboratory of Ophthalmology, Zhongshan Ophthalmic Center, Sun Yat-Sen University, Guangzhou, 510060 People’s Republic of China; 4 Institute of Materia Medica, College of Pharmacy and Laboratory Medicine Science, Army Medical University (Third Military Medical University), Chongqing, 400038 China; 5 Department of Cardiology, Xijing Hospital, Fourth Military Medical University, Xi’an, 710032 China; 6 Department of Hematology, College of Pharmacy and Laboratory Medicine Science, Third Military Medical University (Army Medical University), Chongqing, 400038 China

**Keywords:** Group 2 innate lymphoid cells (ILC2s), Heart, Sex difference, Klrg1, IL-33

## Abstract

**Background:**

Group 2 innate lymphoid cells (ILC2s) are the most dominant ILCs in heart tissue, and sex-related differences exist in mouse lung ILC2 phenotypes and functions; however, it is still unclear whether there are sex differences in heart ILC2s.

**Results:**

Compared with age-matched wild-type (WT) male mice, 8-week-old but not 3-week-old WT female mice harbored an obviously greater percentage and number of heart ILC2s in homeostasis. However, the percentage of killer-cell lectin-like receptor G1 (Klrg1)^−^ ILC2s was higher, but the Klrg1^+^ ILC2s were lower in female mice than in male mice in both heart tissues of 3- and 8-week-old mice. Eight-week-old *Rag2*^*−/−*^ mice also showed sex differences similar to those of age-matched WT mice. Regarding surface marker expression, compared to age-matched male mice, WT female mice showed higher expression of CD90.2 and Ki67 and lower expression of Klrg1 and Sca-1 in heart total ILC2s. There was no sex difference in IL-4 and IL-5 secretion by male and female mouse heart ILC2s. Increased *IL-33* mRNA levels within the heart tissues were also found in female mice compared with male mice. By reanalyzing published single-cell RNA sequencing data, we found 2 differentially expressed genes between female and male mouse heart ILC2s. Gene set variation analysis revealed that the glycine, serine and threonine metabolism pathway was upregulated in female heart ILC2s. Subcluster analysis revealed that one cluster of heart ILC2s with relatively lower expression of Semaphorin 4a and thioredoxin interacting protein but higher expression of hypoxia-inducible lipid droplet-associated.

**Conclusions:**

These results revealed greater numbers of ILC2s, higher expression of CD90.2, reduced Klrg1 and Sca-1 expression in the hearts of female mice than in male mice and no sex difference in IL-4 and IL-5 production in male and female mouse heart ILC2s. These sex differences in heart ILC2s might be due to the heterogeneity of IL-33 within the heart tissue.

**Supplementary Information:**

The online version contains supplementary material available at 10.1186/s12865-022-00525-0.

## Background

Group 2 innate lymphoid cells (ILC2s) are rare but potent ILCs that are involved in allergies and infections by mediating a type 2 immune response [1, 2]. ILC2s are characterized by the expression of the transcription factor Gata3 and the surface markers CD127 (IL-7R), CD90.2 (Thy1.2), and ST2 (IL-33R) (Lin^−^CD127^+^CD90.2^+^ST2^+^). They can produce type 2 cytokines, including interleukin (IL)-4, IL-5, and IL-13 [1, 2]. Our previous study and others have reported that ILC2s are the most dominant population of ILCs in the heart; this population is identified as CD45^+^Lin^−^CD127^+^CD90.2^+^ST2^+^ cells [3, 4]. We found that heart-resident ILC2s have a unique phenotype characterized by lower expression of Icos, CD25 (IL-2Rα), and Ki-67 but higher expression of stem cell antigen 1 (Sca-1) and Gata3 and a stronger ability to produce interleukin (IL)-4 and IL-13 than lung ILC2s [3].

Growing evidence shows that sex differences exist in innate and adaptive immune cells; for instance, male mice have higher NK-cell frequencies in peripheral blood [5], and female mice have higher levels of CD8^+^ T cells and lower levels of regulatory T cells (Tregs) in adipose tissue [6]. Moreover, recent studies have revealed sex-related differences in mouse lung ILC2 phenotypes and functions, which also show strain differences [7]. Heart ILC2s, as a dominant population of ILCs in the heart, have been reported to play a protective role in a mouse model of atherosclerosis [8] and contribute to IL-33-mediated protection of cardiac fibrosis in a mouse model of catecholamine-induced cardiac fibrosis [9]. However, it is still unclear whether there are sex differences in heart ILC2s.

To understand the sex differences in heart ILC2s, we investigated the number, phenotypes, and functions of heart ILC2s in both male and female mice at homeostasis. Here, we showed that male and female mice showed significant differences in the percentage and number of total ILC2s, as well as the Klrg1^+^ and Klrg1^−^ ILC2 subsets, in the heart at 8 weeks old. Compared with male mice, female mouse heart ILC2s exhibited higher expression of CD90.2 and Ki67 and lower expression of Klrg1 and Sca-1; however, there was no difference in IL-4 and IL-5 production in male and female mouse heart ILC2s. As ILC2s are thought to be regulated by regulatory T (Treg) cells that also express IL-33R [10, 11] and sex hormones are known to affect Treg cells, we also used T and B-cell-deficient mice, [6] namely *Rag2*-deficient (*Rag2*^*−/−*^) mice, to verify the sex difference in heart ILC2s. Eight-week-old *Rag2*-deficient (*Rag2*^*−/−*^) mouse heart ILC2s and the Klrg1^+^ and Klrg1^−^ ILC2 subsets exhibited trends similar to those in WT mice. We also found that increased *IL-33* mRNA levels existed within the heart tissues of female mice compared with male mice, which might be responsible for the sex difference in heart ILC2 frequencies and Klrg1 expression. Then, we reanalyzed the single-cell RNA sequencing (scRNA-seq) data and showed 2 differentially expressed genes and differential signaling pathways between male and female mouse heart ILC2s. Heart ILC2s can be divided into 2 clusters, and one cluster with relatively lower expression of Semaphorin 4a (Sema4a) and thioredoxin interacting protein (Txnip) but higher expression of hypoxia-inducible lipid droplet-associated (Hilpda).

## Results

### Female mice show a higher percentage and greater numbers of ILC2s in adult heart tissue

**Fig. 1:**
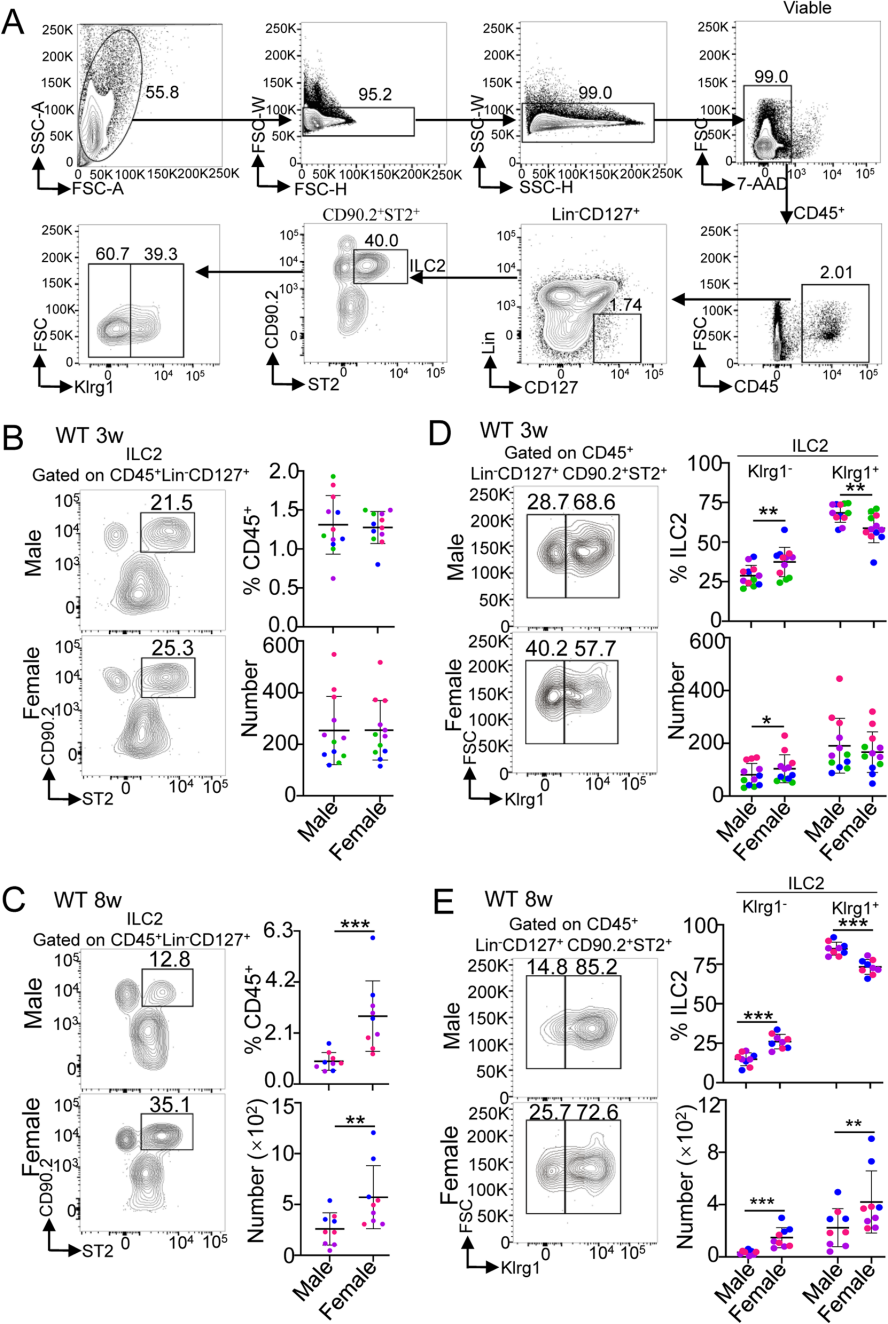
The percentage and numbers of ILC2s in C57BL/6 mouse heart tissue in 3- or 8-week-old wild-type mice. **A** Gate strategy of heart ILC2s in mice. Lineage (Lin) markers included CD3e and CD19. The number inside the gate indicates cell events. **B–C** Cumulative frequencies and enumeration of heart ILC2s among CD45^+^ cells in 3-week-old (**B**) and 8-week-old (**C**) wild-type mice by flow cytometric analysis. **D–E** Cumulative frequencies and enumeration of Klrg1^−^ ILC2s and Klrg1^+^ ILC2s among heart ILC2s in 3-week-old (**D**) and 8-week-old (**E**) wild-type mice by flow cytometric analysis. Each dot represents one mouse; different colors represent different litters; error bars represent the mean ± SD; *p < 0.05, **p < 0.01, ***p < 0.001. Two-way ANOVA followed by Dunnett’s test (**B**–**E**)

To investigate the differences in heart ILC2s between male and female mice, we collected Percoll-enriched heart lymphocytes from 3- and 8-week-old male and female mouse hearts. The gating strategy for heart ILC2s is shown in Fig. 1A. At 3 weeks of age, the percentage and number of heart ILC2s were indistinguishable between male and female mice (Fig. 1B). At 8 weeks of age, both the percentage and the total number of heart ILC2s were higher in female mice than in age-matched male mouse hearts (Fig. 1C).

Based on Klrg1 expression, a recent study reported that the numbers of Klrg1^−^ ILC2s in the bone marrow and lungs are downregulated by androgen [12]. Therefore, we further investigated the sex differences in Klrg1^−^ and Klrg1^+^ ILC2s. The percentages and numbers of heart Klrg1^−^ ILC2s were higher in female mice than in male mice at the age of both 3 and 8 weeks when CD45^+^Lin^−^CD127^+^CD90.2^+^ST2^+^ ILC2s were assessed. For heart Klrg1^+^ ILC2s, the percentage was decreased, but the numbers were increased in 8-week-old mice, but a decreased percentage and a decreasing trend in the number of heart Klrg1^+^ ILC2s were found at 3 weeks of age (Fig. 1D–E).

In addition, we also measured the protein levels of Gata3 in heart ILC2s and found that CD45^+^Lin^−^CD127^+^CD90.2^+^ST2^+^ cells showed over 90% Gata3 expression, and the percentage and numbers of heart CD45^+^Lin^−^CD127^+^CD90.2^+^ST2^+^Gata3^+^ cells [9] were also higher than those in male mice (Additional file 1: Fig. S1A). To further illustrate heart ILC2s exist sex difference, we also gated CD45^+^Lin^−^CD90.2^+^CD127^+^Gata3^+^ for ILC2 as done in another published article [13], and the data showed that the percentage of ILC2s among CD45^+^ cells were higher in female mice than male mice (Additional file 1: Fig. S1B). The above data suggest that sex hormones may play an extrinsic role in determining the number of heart ILC2s, especially the numbers of Klrg1^−^ ILC2s and Klrg1^+^ ILC2s in the heart.

### Phenotypic differences in heart ILC2s between male and female mice

**Fig. 2:**
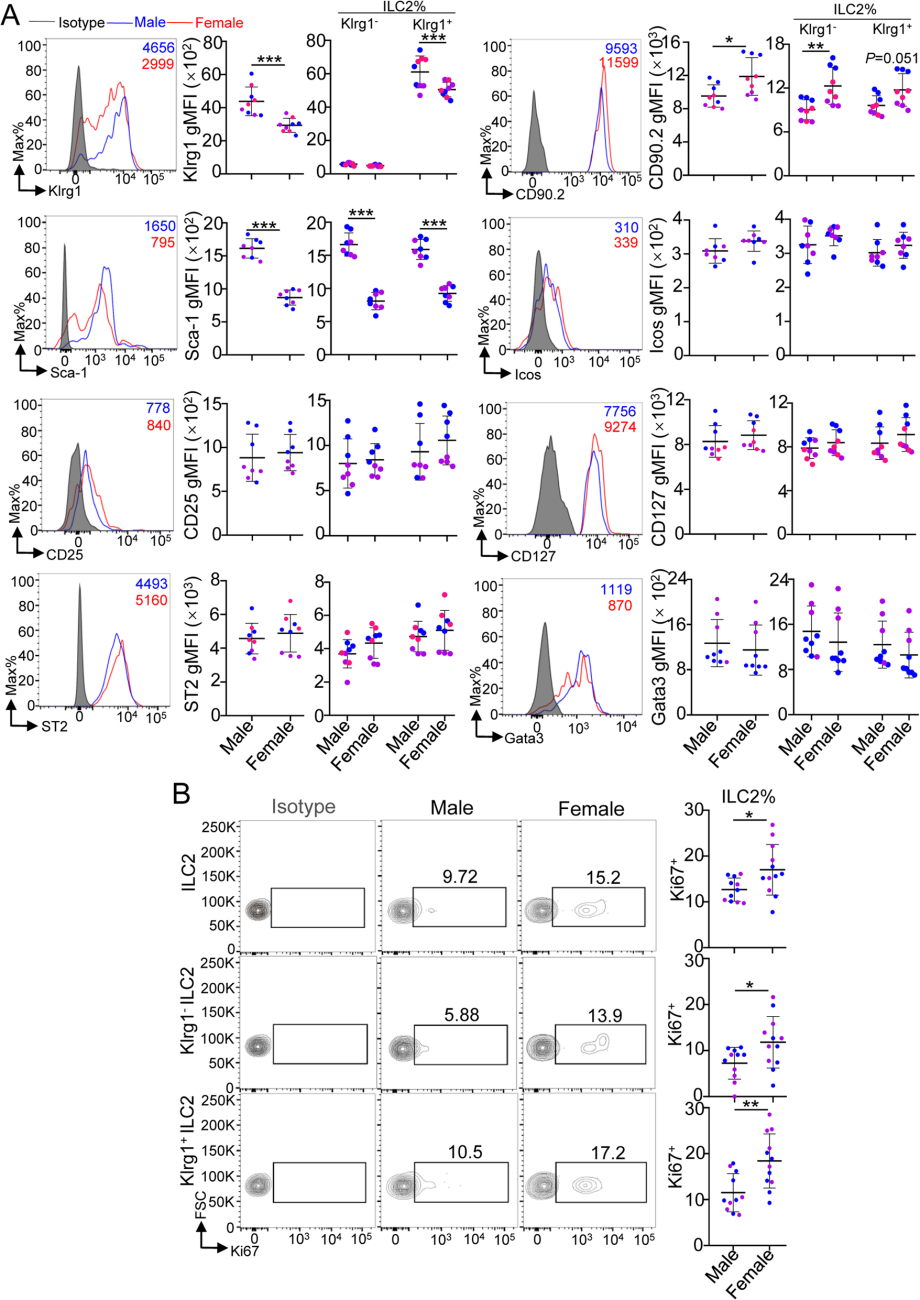
Phenotypic differences between male and female mouse heart ILC2s. **A** The geometric mean fluorescence intensity (gMFI) of the indicated surface markers in heart ILC2s (identified as CD45^+^Lin^−^CD127^+^CD90.2^+^ST2^+^ cells) and heart Klrg1^−^ ILC2s and Klrg1^+^ ILC2s of 8-week-old mice. **B** The cumulative frequencies of Ki67 expression in heart ILC2s and Klrg1^−^ ILC2s and Klrg1^+^ ILC2s of 8-week-old mice by flow cytometric analysis. Each dot represents one mouse; different colors represent different litters; error bars represent the mean ± SD; *p < 0.05, **p < 0.01. Two-way ANOVA followed by Dunnett’s test (**A**–**B**)

We next investigated the sex differences in murine heart ILC2 phenotypes, including surface markers, transcription factors, and proliferation. In total heart ILC2s, the geometric mean fluorescence intensity (gMFI) of Klrg1 and Sca-1 was lower, while CD90.2 (Thy1.2) was higher in 8-week-old female mice than in age-matched male mice (Fig. 2A). There were no sex differences in the gMFIs of other surface markers, including Icos, CD25 (IL-2Rα), CD127 (IL-7R) and ST2 (IL-33R), or in that of the transcription factor Gata3 (Fig. 2A). In addition, female mouse heart ILC2s had a stronger proliferation ability than male mice, which was reflected by increased Ki67^+^ cells (Fig. 2B). These results demonstrated that mouse heart total ILC2s had distinct sex differences in terms of the surface expression of Klrg1, Sca-1, CD90.2 and proliferation ability.

Because our results showed that the numbers of Klrg1^−^ ILC2s and Klrg1^+^ ILC2s in the heart exhibited sex differences, we next determined the sex differences in heart Klrg1^−^ or Klrg1^+^ ILC2 phenotypes. Our results showed that the gMFI of CD90.2 was increased, and the gMFI of Sca-1 was decreased in both Klrg1^−^ and Klrg1^+^ ILC2s in the heart of female mice, but the gMFI of Klrg1 was also decreased in Klrg1^+^ ILC2s in female mouse hearts (Fig. 2A). The number of Ki67-positive cells was higher in both Klrg1^−^ ILC2s and Klrg1^+^ ILC2s in female mouse hearts (Fig. 2B). There were no sex differences in CD25, Icos or Gata3 among heart Klrg1^−^ ILC2s and Klrg1^+^ ILC2s (Fig. 2A).

### Cytokines secreted by heart ILC2s in male and female mice

**Fig. 3:**
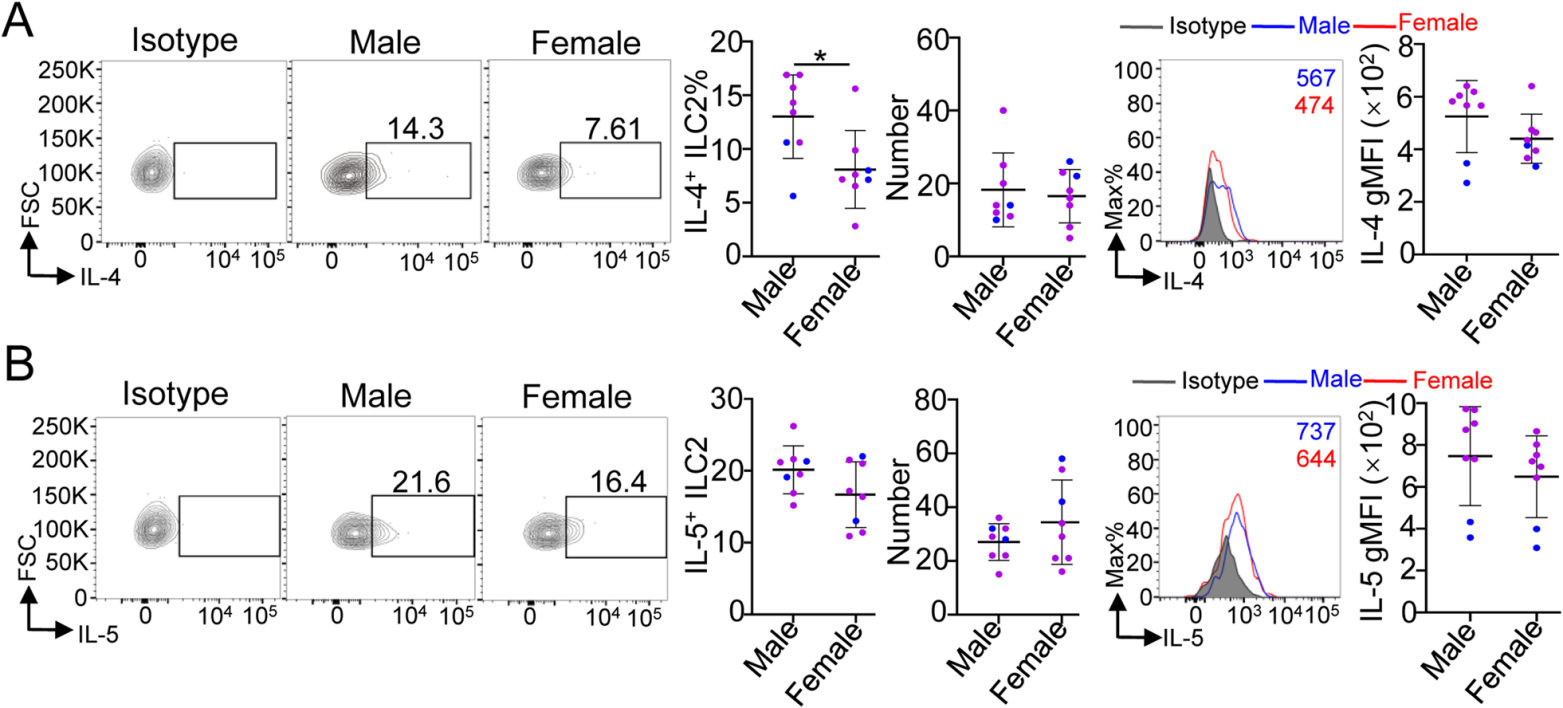
Cytokine production ability of heart ILC2s in 8-week-old mice. **A–B** The cumulative frequencies, enumeration and gMFI of IL-4 (**A**) and IL-5 (**B**) in the heart to ILC2s after IL-33 stimulation for 4 h by flow cytometric analysis. Each dot represents one mouse; different colors represent different litters; error bars represent the mean ± SD. *p < 0.05. Unpaired two-tailed Student’s t test (**A**–**B**)

ILC2s are known to produce the main type 2 cytokines, IL-4 and IL-5, after stimulation with IL-25, IL-33 and TSLP. To investigate the expression of these cytokines in male and female mouse heart ILC2s, we stimulated heart lymphocytes with 50 ng/ml IL-33 for 4 h and then determined the production of IL-4 and IL-5 by the ILC2s through flow cytometry. Our results showed that heart ILC2s showed a lower percentage of IL-4 in female mice than in age-matched male mice (Fig. 3A), but the total number of IL-4^+^ ILC2s and the gMFI of IL-4 exhibited no significant difference between male and female mouse heart ILC2s (Fig. 3A). There was no significant difference in IL-5 production, regarding the percentage, total number and gMFI, in heart ILC2s between male and female mice (Fig. 3B). The above data suggest that IL-4 and IL-5 secreted by heart ILC2s are not affected by sex hormones.

### The number and percentage of heart ILC2s are different between male and female *Rag2*^*−/−*^ mice

**Fig. 4:**
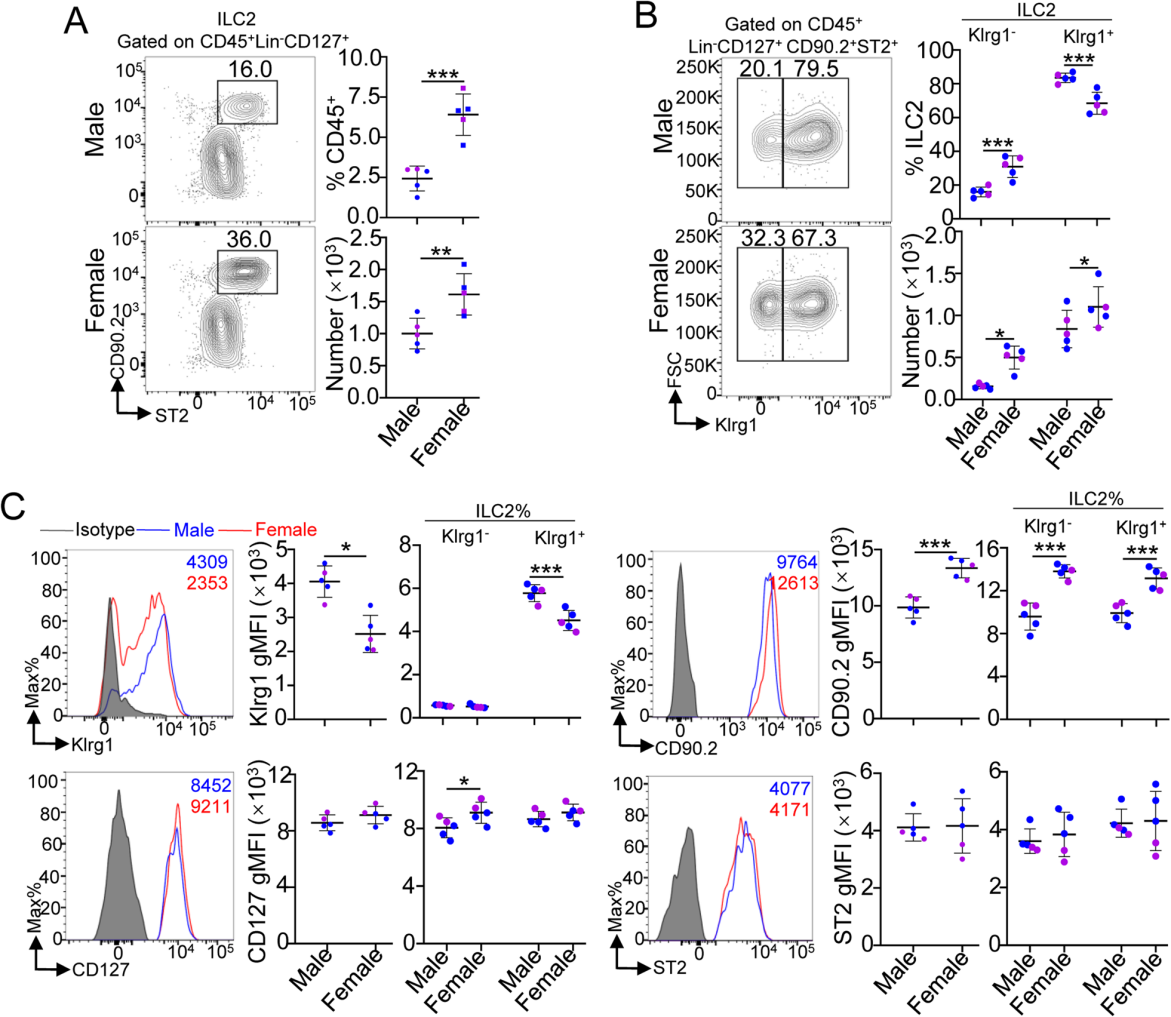
Percentage and cell numbers of ILC2s in the heart tissues of 8-week-old *Rag2*^*−/−*^ mice. **A** Cumulative frequencies and enumeration of heart ILC2s among CD45^+^ cells in 8-week-old *Rag2*^*−/−*^ mice. **B** Cumulative frequencies and enumeration of Klrg1^−^ ILC2s and Klrg1^+^ ILC2s among heart ILC2s in 8-week-old *Rag2*^*−/−*^ mice. **C** The gMFIs of the indicated surface markers in heart ILC2s (identified as CD45^+^Lin^−^CD127^+^CD90.2^+^ST2^+^ cells) and heart Klrg1^−^ ILC2s and Klrg1^+^ ILC2s of 8-week-old *Rag2*^*−/−*^ mice. Each dot represents one mouse; error bars represent the mean ± SD; **p < 0.01, ***p < 0.001. Two-way ANOVA followed by Dunnett’s test (**A**–**C**)

ILC2s are thought to be regulated by Treg cells that also express ST2 [10, 11], and sex hormones are known to affect Treg cells [6]. As such, we also determined the percentage and number of heart ILC2s in male and female *Rag2*^*−/−*^ mice, which lack functional T and B cells, at the age of 8 weeks old. The data showed a higher percentage and number of total heart ILC2s in female mice than in male mice (Fig. 4A). The percentages and numbers of heart Klrg1^−^ and Klrg1^+^ ILC2s between male and female *Rag2*^*−/−*^ mice showed trends similar to those observed for WT mice (Fig. 4B). Interestingly, the number of total heart ILC2s in *Rag2*^*−/−*^ mice was almost threefold higher than that in WT mice (male mice: 258.89 ± 157.84 vs. 1000.6 ± 220.18; female mice: 570.11 ± 310.08 vs. 1612.00 ± 321.78). The increased number of heart ILC2s in *Rag2*^*−/−*^ mice was mainly contributed by Klrg1^−^ ILC2s. This difference might be attributed to Treg cells, partly because Treg cells repress ILC2 functions directly or compete for IL-33 in heart tissue [10].

Because our results showed that the phenotypes of heart ILC2s and Klrg1^−^ ILC2s and Klrg1^+^ ILC2s, including Klrg1 and CD90.2, were different in male and female WT mice, we also determined the sex difference of heart Klrg1^−^ or Klrg1^+^ ILC2s in phenotypes. Our results showed that the gMFI levels of Klrg1 were lower but CD90.2 was higher in female *Rag2*^−/−^ mice (Fig. 4C), which was similar in WT mice. Moreover, the gMFI level of CD90.2 were increased in both subsets of female mice heart, but the gMFI level of Klrg1 were decreased in female mouse heart Klrg1^+^ILC2s, and the gMFI level of CD127 was higher in only female mouse heart Klrg1^−^ ILC2s (Fig. 4C). There was no sex difference in ST2 among heart Klrg1^−^ ILC2s and Klrg1^+^ ILC2s (Fig. 4C).

### Expression of hormone receptors on heart ILC2s and IL-33 in heart tissue

**Fig. 5:**
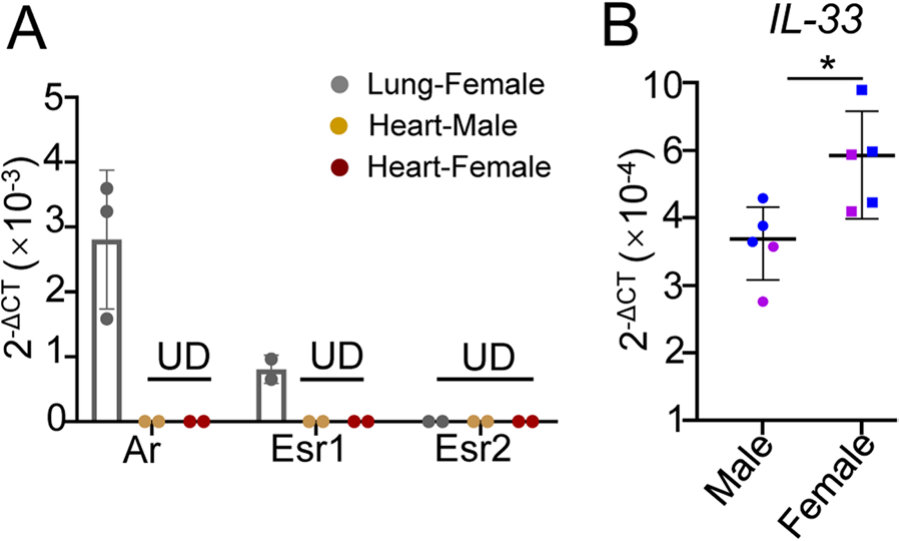
The levels of hormone receptors on heart ILC2s and IL-33 in the heart tissue in both male and female mice. **A** The relative mRNA expression of *Ar*, *Esr1*, and *Esr2* in both lung and heart ILC2s. **B** The relative mRNA expression of *IL-33* in the heart tissues of both male and female mice. Each dot represents one mouse; different colors represent different litters; error bars represent the mean ± SD. *p < 0.05. Unpaired two-tailed Student’s t test (**A**–**B**)

We further explored whether the sex differences in heart ILC2s are dependent on cell intrinsic sex hormone receptor expression levels or extrinsic cytokines from local heart tissues. Previous studies have found that lung ILC2s showed higher expression levels of androgen receptor (Ar), lower expression of estrogen receptor 1 (Esr1) and no expression of Esr2 [14], and sex hormones, such as androgen, play an extrinsic role in determining the numbers of lung ILC2s and ILC2 progenitors [15, 16]. To this end, we measured the mRNA expression of *Ar*, *Esr1* and *Esr2* in both male and female mouse heart ILC2s and found that the mRNA levels of *Ar*, *Esr1* and *Esr2* were undetectable in both male and female mouse heart ILC2s (Fig. 5A). Consistently, the percentages and surface marker expression of heart ILC2s from both male and female mice were not changed after stimulation with different concentrations of 17β-E2 (estrogen) or testosterone (androgen) for 12 h (Additional file 1: Fig. S2A-S2D). Interestingly, there was no significant difference between Klrg1, CD90.2 and Sca-1 expression levels in heart ILC2s between female and male mice after in vitro coculture. This suggests a cell-extrinsic factor existed in the heart tissue may contribute to the sex differences of heart ILC2s.

To further explore the extrinsic factors, such as cytokines, that respond to sex differences in heart ILC2s, we determined the mRNA levels of *IL-33*, which is reported to maintain ILC2 homeostasis and expansion in the heart tissues of both male and female mice in a homeostatic state [9, 17, 18]. As IL-33 is mainly expressed by nonhematopoietic cells, such as fibroblasts, epithelial cells, and endothelial cells [19], we used whole heart tissue for RNA preparation. The data revealed relatively higher mRNA levels of IL-33 in female mouse heart tissue than in male mouse heart tissue (Fig. 5B). Collectively, these data suggested that the sex difference in heart ILC2s is not dependent on sex hormone receptor expression but might be associated with the higher levels of IL-33 within the whole heart tissue of female mice.

### Single-cell transcriptional profiles of heart ILC2s in male and female mice

**Fig. 6:**
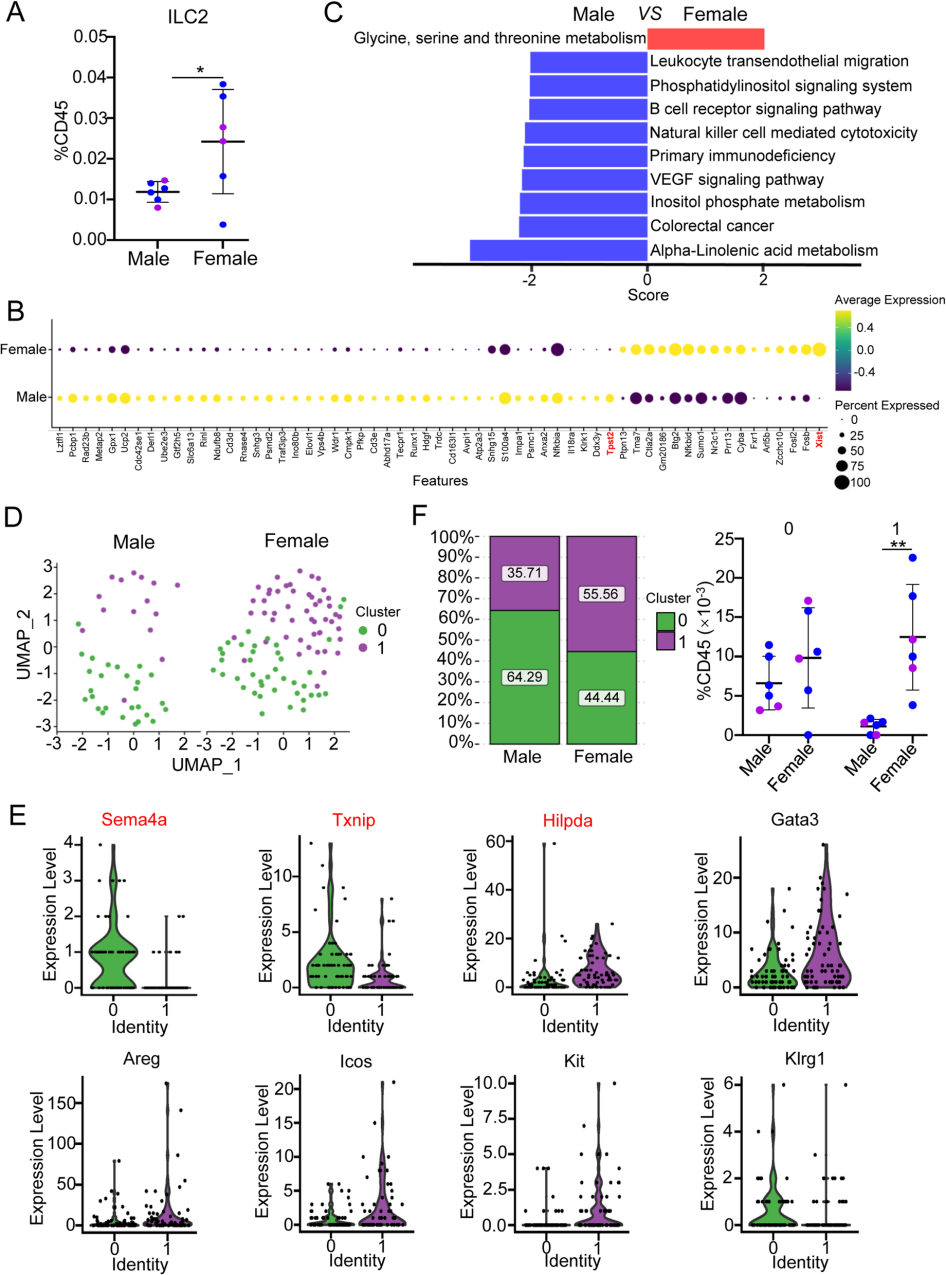
RNA-Seq analysis of heart lymphocytes from both male and female mice. **A** Percentage of ILC2s relative to CD45^+^ cells in heart tissues of both male and female mice. The percentage of heart ILC2s among CD45^+^ cells in female or male mouse for each sample is the cell numbers of female or male heart ILC2s divided by the total numbers of corresponding female or male CD45^+^ cells, respectively. Each dot represents one sample; error bars represent the mean ± SD; *p < 0.05; unpaired two-tailed Student’s t test. **B** Bubble plot representing the relative abundance of differentially expressed genes in heart ILC2s between male and female mice. DEGs are highlighted by red. The logFC threshold was set to 0.25 and adjust *P* value was set to < 0.05, respectively. **C** Pathway enrichment assay of highly expressed genes in both male and female heart ILC2s by GSVA. **D** UMAP reduction and data visualization of heart ILC2s. Two clusters of heart ILC2s were divided after unsupervised clustering. **E** Violin plots showing the expression of marker genes in 2 clusters of heart ILC2s, and DEGs are highlighted by red. **F** The percentages of Clusters 0 and 1 in male and female mouse heart ILC2s (left) and the percentage of Clusters 0 and 1 relative to CD45^+^ cells in male and female mouse hearts (right)

To further explore the sex difference of heart ILC2s at the single-cell level, we reanalyzed the single-cell RNA sequencing (scRNA-seq) data by using two published datasets together, one containing 2 [20] and one containing 4 cardiac nonmyocytes samples [21], both of which were pooled from one paired WT female and male heart tissue, for scRNA-seq. After initial quality control checks, a total of 5793 CD45^+^ cells were acquired and subdivided into 8 main clusters based on marker gene expression across all cells by uniform manifold approximation and projection (UMAP) analysis (Additional file 1: Fig. S3A). Cell plots with red circles were attributed to ILC2s, which was confirmed by the high expression of *Gata3* and *Il7r* (Additional file 1: Fig. S3A-S3B). Because these scRNA-seq samples were mixtures of cells derived from both male and female mice, we isolated male and female CD45^+^ cells based on the expression of the female-specific gene *Xist* (X-inactive specific transcript) [21] (Additional file 1: Fig. S3C). The data showed relatively higher percentages of ILC2s among CD45^+^ cells in female mice than in male mice (Fig. 6A), which is consistent with our flow cytometric results. Due to the limited cell numbers of heart ILC2s acquired from the published data, we only identified Xist and Tyrosylprotein sulfotransferase 2 (Tpst2) as differentially expressed genes (DEGs) in heart ILC2s from male and female mice (adjust *P* < 0.05) (Fig. 6B and Additional file 2: Table S1). Although without statistical significance, DEAD-Box helicase 3 Y-linked (Ddx3y) (30.8% of total male ILC2), Killer cell lectin like receptor K1 (Klrk1) (25.6% of total male ILC2s), and Il18 receptor 1 (Il18r1) (25.6% of total male ILC2s) were only detected in male ILC2s, while Fragile X mental retardation autosomal homolog 1 (Fxr1) (27.4% of total female ILC2s) and ADP ribosylation factor like GTPase 5b (Arl5b) (33.9% of total female ILC2s) were mainly detected in female ILC2s (Additional file 2: Table S1). Gene set variation analysis (GSVA) revealed that the glycine, serine and threonine metabolism pathway was upregulated, while other signaling pathways, such as the B-cell receptor signaling pathway and alpha-linolenic acid metabolism, were downregulated in female heart ILC2s compared with male heart ILC2s (Fig. 6C).

To explore the heterogeneity of heart ILC2s in both female and male mice, heart ILC2s were further subdivided into 2 clusters, Cluster 0 and 1 (Fig. 6D). Due to the limited cell numbers of heart ILC2s acquired from the published data, only 3 DEGs were found between the two clusters after adjusting *P* values, including Semaphorin 4a (Sema4a), Thioredoxin interacting protein (Txnip), and Hypoxia-inducible lipid droplet-associated (Hilpda) (adjust *P* < 0.05) (Additional file 2: Table S2). Cluster 1 showed relatively lower expression of Sema4a and Txnip and higher expression of Hilpda than Cluster 0 (Fig. 6E). In addition, Cluster 1 also showed relatively higher expression of Gata3, Areg, Icos, and Kit but lower expression of Klrg1 than Cluster 0, although without statistical significance (Fig. 6E). The percentages of Clusters 0 and 1 were 35.71% and 64.29%, respectively, in male heart ILC2s and 55.56% and 44.44%, respectively, in female mouse heart ILC2s (Fig. 6F left panel). After calculating the percentage of Cluster 0 and 1 heart ILC2s among CD45^+^ cells, we found that the percentage of Cluster 0 heart ILC2s among CD45^+^ cells showed no difference between female and male mice, whereas the percentage of Cluster 1 heart ILC2s among CD45^+^ cells was obviously higher in female mice than in male mice (Fig. 6F right panel). Due to limited cell numbers of heart ILC2s in this dataset, we did not find any DEGs after adjusting *P* values between male and female mice in cluster 0 and 1 except Xist (Additional file 2: Table S3-S4). Although without statistical significance, 149 genes were mainly detected in cluster 0 of female, including C–C motif chemokine ligand 3 (Ccl3) (25% of female in cluster 0) (Additional file 2: Table S3) that has been reported to have sex difference in the hippocampus of rats [22]. And 26 genes were only detected in cluster 1 of female, such as Peroxisome proliferator activated receptor gamma (Pparg) (22% of female in cluster 1) (Additional file 2: Table S4), which has been found that Pparg-regulated genes were upregulated in female adipose tissue compared with male adipose [23]. These findings suggest that the higher percentage and greater numbers of ILC2s in female heart tissue are mainly due to more Cluster 1 cells.

## Discussion

Recent studies have highly demonstrated tissue- and even strain-specific sex differences in resident ILC2s [12, 15, 24]. The frequency and numbers of ILC2s were found to be higher in the visceral adipose tissue, mesenteric lymph nodes and lungs of adult female mice than in those of male mice under steady-state conditions [12, 15]. We and other groups have reported that ILC2s are the most dominant population of ILCs in the heart [3, 4]. In the current study, we found that the percentage and number of total ILC2s in the heart were significantly higher in female mice than in male mice in adulthood but not at 3 weeks old in the steady state. In addition, female and male *Rag2*^*−/−*^ mice showed similar trends for the percentage and numbers of total ILC2s and Klrg1 expression.

In female C57BL/6 mice, lung ILC2s exhibited increased expression of CD90.2 and decreased expression of Klrg1 [7, 12, 15]. Similarly, our current study showed that female mouse heart ILC2s exhibited higher expression levels of CD90.2 but lower expression of Klrg1 and Sca-1 at 8 weeks of age. Klrg1 is expressed on ILC2s, natural killer (NK) cells and CD8^+^ T cells and is an inhibitory receptor that contains an immunoreceptor tyrosine-based inhibitory motif (ITIM) [25–27]. Recent reports have revealed that Klrg1 interacts with E-cadherin in epithelial tissue to blunt mouse lung ILC2 proliferation [28, 29]. Consistent with this scenario, our data showed that female heart Klrg1^+^ ILC2s showed a reduced MFI for Klrg1 along with increased Ki67 expression. Consistently, our scRNA-seq analysis showed that female mouse heart ILC2s had relative higher expression of Fxr1 and Arl5b, though without statistical significance. Fxr1 is RNA-binding protein, which promotes cancer cell proliferation, including prostate cancer cells [30] and ovarian cancer cells [31], whereas male mouse heart ILC2s showed upregulation of α-linolenic acid metabolism, which decreases T-cell proliferation and differentiation [32].

Previous studies have demonstrated that sex differences in ILC2s are regulated by both sex hormone receptor-dependent and -independent pathways [14]. In our study, murine heart ILC2s expressed almost undetectable sex hormone receptors, and heart ILC2s showed no sex differences in response to sex hormone stimulation, which suggests that the sex difference in murine heart ILC2s is not dependent on sex hormones. However, our results revealed a relatively higher mRNA level of IL-33 in the heart tissue of female mice than in male mice at a steady state. Previous studies have demonstrated that IL-33 enhances ILC2 percentages at steady state [17]. All this evidence suggests that the heterogeneity of IL-33 levels in the heart tissue may contribute to the increased percentage of heart ILC2s in female mice at a steady state.

Previous studies have shown that ILC2s are a heterogeneous population based on their differential responses to the microenvironment in the lung [33], skin [34] and small intestine [35]. It can be subdivided into inflammatory ILC2s (iILC2s) responding to IL-25 or helminthic infection and natural ILC2s (nILC2s) responding to IL-33 [33, 36]. Although there were only 101 ILC2 cells, we could still subdivide heart ILC2s into 2 clusters, Clusters 0 and 1. The percentage of Cluster 1, but not Cluster 0, heart ILC2s among CD45^+^ cells in each heart were obviously higher in female mice than in male mice, which was responsible for the higher percentage and greater numbers of ILC2s in female heart tissue. Cluster 1 showed relatively lower expression of Sema4a and Txnip and higher expression of Hilpda than Cluster 0. Additionally, Cluster 1 showed lower expression of Klrg1 but higher expression of GATA3, although without statistical significance, which might be due to the limited cell numbers we acquired from the published data. Sema4a is a Semaphorin that functions in angiogenesis, tumor and immune responses [37] and plays an important role in T-cell activation and the balance between Th1 and Th2 cell responses [38, 39]. Txnip, a thioredoxin (TRX)-binding protein, is involved in the production of IFN-γ in natural killer cells [40] and is associated with nod-like receptor protein 3 (NLRP3) inflammasome activation [41]. Hilpda, an oncogenic gene, is widely expressed in various tumors and is related to macrophage infiltration in the tumor microenvironment [42]. However, we did not find any differentially expressed genes between male and female mice in cluster 0 and 1 due to the limited cell numbers of heart ILC2s we used. Therefore, the detailed surface marker and functional and sex difference between Clusters 0 and 1 should be further discussed, which warrants another separate study.

## Conclusions

In summary, the results of the present study present sex differences in adult murine heart ILC2s, characterized by female mice harboring obviously greater numbers of heart ILC2s in homeostasis due to a major subset of Klrg1^−^ ILC2s and no sex difference in IL-4 and IL-5 production, which might be because of the heterogeneity of IL-33 levels within the heart tissue. By reanalyzing the published scRNA-seq data, heart ILC2s can be subdivided into 2 clusters. The percentage of Cluster 1 heart ILC2s among CD45^+^ cells in each heart was obviously higher in female mice, which is a response to the higher percentage and greater numbers of ILC2s in female heart tissue.

## Methods

### Animals

Male and female wild-type (WT) C57BL/6 mice were maintained in the Hunan Children’s Research Institute pathogen-free animal facility of Hunan Children’s Hospital (Changsha, Hunan, China). Mice were housed in cages (4–5 mice maximum per cage) at 22–25 °C and 50 ± 10% relative humidity with a 12-h light/dark cycle, periodic air changes, and free access to water and food. Congenic *Rag2*^*−/−*^ mice on the C57BL/6 background were obtained from Changzhou Cavens Laboratory Animal Co., Ltd. (Changzhou, Jiangsu, China). All animal procedures and protocols were approved by the Animal Ethics Committee of Hunan Children’s Hospital and followed the guidelines of the Institutional Animal Care and Use Committees of Hunan Children’s Hospital (Changsha, Hunan, China).

### Single mononuclear cell suspension preparation

A single-cell suspension was prepared as described previously [3]. Mice were anesthetized with 2% pentobarbital sodium, and the heart was slowly perfused with cold phosphate-buffered saline (PBS) administered via the left ventricle with a 5-ml syringe to remove peripheral blood cells. Then, heart tissues were cut into approximately 1-cm^2^ pieces and digested for 45 min at 37 °C in Hank’s solution containing 10% fetal bovine serum (FBS) (Biological Industry, Kibbutz Beit Hemek, Israel), 0.5 mg/ml collagenase I (Sigma‒Aldrich, St. Louis, MO, United States), and 0.5 mg/ml collagenase II (Gibco, Waltham, MA, United States). After digestion, the cells were resuspended in 20% Percoll (GE Healthcare, Pittsburgh, PA, United States) in RPMI 1640 medium (Biological Industry, Kibbutz Beit Haemek, Israel) containing 5% FBS, and a single mononuclear cell suspension was collected after centrifugation (2000 rpm, room temperature, 5 min).

### Antibodies and flow cytometry

The antibodies used for flow cytometry were commercially purchased and are listed in Table 1. For surface markers, single-cell suspensions derived from heart tissues were stained by incubating the cells with antibodies in staining buffer (PBS containing 2% mouse serum, 2% horse serum and anti-CD16/CD32 blocking antibodies (eBioscience, San Diego, CA, United States)) for 15 min at room temperature in the dark. For live-dead staining, cells were incubated with 7-AAD in apoptosis staining buffer (BioLegend, San Diego, San Diego, CA, United States) for 15 min at 4 °C after surface marker staining. Gata3 and Ki67 were stained as recommended by the manufacturer using the Foxp3/Transcription Factor Staining Buffer Set Kit (eBioscience, San Diego, CA, United States). The lineage (Lin) markers included CD3ε and CD19. Isotype-matched control antibodies were used at the same concentration as the corresponding test antibody. All flow cytometry experiments were carried out on a BD LSRFortessa (BD Biosciences, San Diego, CA, United States). Data were analyzed with FlowJo software (version 10.0; FlowJo LLC, Ashland, OR United States).

**Table 1:** Antibodies used for flow cytometry

Antibodies	Clone	Source	Dilution
Anti-mouse CD45	30-F11	BioLegend	1/200
Anti-mouse CD3ε	145-2C11	BioLegend	1/200
Anti-mouse CD19	6D5	BioLegend	1/200
Anti-mouse CD127	SB/199	BD Bioscience	1/100
Anti-mouse CD90.2	53–2.1	BioLegend	1/100
Anti-mouse ST2	U29-93	BD Bioscience	1/100
Anti-mouse Klrg1	2F1	BioLegend	1/100
Anti-mouse Icos	7E.17G9	BioLegend	1/100
Anti-mouse CD25	UC10-4B9	BioLegend	1/100
Anti-mouse Sca-1	D7	BioLegend	1/100
Anti-mouse Gata3	16E10A23	BioLegend	1/20
Anti-mouse IgG2b	MPC-11	BioLegend	1/100
Anti-mouse Ki67	SolA15	Thermo-eBioscience	1/100
Anti-mouse IgG2a	eBR2a	Thermo-eBioscience	1/100
Anti-mouse IL-4	11B11	BD Bioscience	1/50
Anti-mouse IgG1	R3-34	BD Bioscience	1/50
Anti-mouse IL-5	TRFK5	Thermo-Invitrogen	1/50
Anti-mouse IgG1	eBRG1	Thermo-Invitrogen	1/50

### RNA isolation and qRT‒PCR analysis

For analysis of the sex hormone receptor of heart ILC2s, single mononuclear cell suspensions were isolated from male and female mouse heart tissues according to the above description, and heart ILC2s were sorted by fluorescence-activated cell sorting (FACS) using a FACSAria III cell sorter (BD Biosciences, San Jose, CA, USA) after gating on CD45^+^Lin^−^CD127^+^CD90.2^+^ST2^+^ cells. The purity of heart ILC2s was measured with the gating strategy (Additional file 1: Fig. S4). Then, the sorted heart ILC2s with purity > 90% were used for RNA extraction with an RNAprep Pure Micro Kit (Tiangen Biotech, Beijing, China). To analyze the expression of IL-33 in heart tissue, total RNA was extracted from whole heart tissue from male and female mice using TRIzol (Invitrogen, Waltham, MA, United States), as described previously [3].

Total RNA was then reverse transcribed into cDNA using *Evo M-MLV* RT Premix (AG Biotechnology (Hunan), Changsha, China). Real-time qPCR was performed using a SYBR® Green Premix *Pro Tag* HS qPCR Kit (AG Biotechnology (Hunan), Changsha, China) with a Roche LightCycler® 480 II. Primer sequences used for qRT‒PCR were obtained from reported literature or designed by Pubmed Primer-BLAST. Primer sequences used for qRT‒PCR were designed by PubMed Primer-BLAST, including Ar forward, 5′-CAGGAGGTAATCTCCGAAGGC-3′; Ar reverse, 3′-ACAGACACTGCTTTACACAACTC-5′; Esr1 forward, 5′-CCCGCCTTCTACAGGTCTAAT-3′; Esr1 reverse, 3′-CTTTCTCGTTACTGCTGGACAG-5′; Esr2 forward, 5′-CTGTGATGAACTACAGTGTTCCC-3′; Esr2 reverse, 3′-CACATTTGGGCTTGCAGTCTG -5′. Primer sequences used for qRT‒PCR were obtained from the reported literature, including IL-33 forward, 5′-CCCTGGTCCCGCCTTGCAAAA-3'; IL-33 reverse, 3′-AGTTCTCTTCATGCTTGGTACCCGA-5′ [3]; GAPDH forward, 5′-AGGTCGGTGTGAACGGATTTG-3′; GAPDH reverse, 3’ TGTAGACCATGTAGTTGAGGTCA-5′.

### Analysis of sex hormone response of heart ILC2s

For the analysis of the heart ILC2 response to sex hormones, single mononuclear cell suspensions isolated from heart tissues were stimulated with 0, 0.1, 1 and 10 nM 17β-estradiol (17β-E2) and testosterone (Medchem Express, Monmouth Junction, NJ, United States) supplemented with 10 ng/mL IL-2 and IL-7 (BioLegend, San Diego, San Diego, CA, United States) to survival for 12 h [9] and then stained for surface markers. To rule out sex hormone effects from the culture medium and FBS, we used charcoal dextran stripped FBS (Serana Europe GmbH, Brandenburg, Germany) and phenol red free culture medium (Boster Biological Technology, Wuhan, China) in this experiment. All flow cytometry experiments were carried out on a BD LSRFortessa. Data were analyzed with FlowJo software.

### IL-4 and IL-5 production

For intracellular IL-5 and IL-4 staining, single-cell suspensions isolated from heart tissues were stimulated with 50 ng/ml IL-33 (BioLegend, San Diego, San Diego, CA, United States) plus BD Golgi Plug protein transport inhibitor (BD Biosciences, San Diego, CA, United States) for 4 h and then stained for surface markers. After washing, the cells were fixed with the Fixation/Permeabilization Solution Kit (BD Biosciences, San Diego, CA, United States) following the manufacturer’s instructions and stained with anti-IL-4 or anti-IL-5 antibodies. Isotype-matched control antibodies were used at the same concentration as the corresponding test antibody. All flow cytometry experiments were carried out on a BD LSRFortessa. Data were analyzed with FlowJo software.

### scRNA-seq analysis of heart ILC2s

A total of 6 cardiac nonmyocyte samples pooled from one paired WT female and male mouse heart tissue were downloaded from the ArraryExpress database [20, 21] for downstream analysis. Cell Ranger version (version 6.0.1) was used to process raw sequencing data. The Seurat R package (version 4.0.5) was applied in our study to convert the scRNA-seq data as a Seurat object [43]. Cells that expressed fewer than 300 genes or more than 5000 genes or more than 20% mitochondrial genes were removed at the quality control step. Data were then normalized by the SCT transform R package (version 0.3.2) [44]. Next, we used the “RunPCA” function to reduce the dimensionality of the scRNA-seq data. Subsequently, we used the “RunPCA” function to conduct UMAP analysis. We also used the “Find Clusters” and “Find All Markers” functions to conduct cell clustering analysis and detect gene expression markers. Afterward, we used the Single R package and Cell Marker dataset to annotate the cell types in our study [45]. The “subset” function was also applied to extract the subcluster for downstream analysis and then annotated and analyzed as described above. Cells with Xist gene expression ≥ 0.1 were identified from female mice. To analyze the DEGs between female and male ILC2s, the value of the logFC threshold was set to 0.25 to filter the DEGs (Adjust *p* value < 0.05), and the heatmap was produced by the Complex Heatmap R package (version 2.11.1). A total of 186 KEGG pathway were download from the Molecular Signatures Database (MSigDB, http://www.gsea-msigdb.org/gsea/msigdb/index.jsp) by the package “msigdbr”, with the permission from Kyoto Encyclopedia of Genes and Genomes (KEGG, https://www.kegg.jp/) website [46–49]. GSVA analysis was performed to assess these KEGG pathway activation in the GSVA R package [50]. The parameters of msigdbr were set as follows: species = “Mus musculus”, category = “C2”, subcategory = “KEGG”.

### Statistical analysis

All data are expressed as the mean ± SD, and statistical analyses were performed with SPSS software for Windows (Version 23, SPSS Inc., Chicago, IL, United States). Statistical analysis was performed with an unpaired Student’s t test for comparisons of two independent experimental groups. If the litter effect was very obvious among independent experiments, two-way ANOVA (sex and litter) followed by Dunnett’s test was used [51]. In each analysis, there were n = 5–12 replicates per group, and the results are representative of at least two independent experiments. Statistical significance was defined as *p* < 0.05. The number of mice used in each group is indicated in the figure legends. All graphs were produced by GraphPad Prism 8.0 for Windows software (GraphPad Software Inc., La Jolla, CA, United States).

## Supplementary Information

**Additional file 1**. **Figure S1**: The percentage and numbers of heart Gata3^+^ ILC2s in 8-week-old wild-type C57BL/6 mice. A Cumulative frequencies and enumeration of heart Gata3^+^ ILC2s among ILC2s (identified as CD45^+^Lin^-^CD127^+^CD90.2^+^ST2^+^ cells, left) or CD45^+^ lymphocytes (middle) in 8-week-old wild-type mice by flow cytometric analysis. B Cumulative frequencies and enumeration of heart GATA3^+^CD127^+^ ILC2s among CD45^+^ lymphocyte in 8-week-old wild type mice by flow cytometric analysis. Each dot represents one mouse; different colors represent different litters; error bars represent the mean ± SD; *p < 0.05, **p < 0.01. Unpaired two-tailed Student’s t test (A-B). **Figure S2:** The responsiveness of heart ILC2s to sex hormones. A-B The cumulative frequencies of heart ILC2s among CD45+ cells treated with the indicated concentrations of 17β-E2 (A) and testosterone (B) for 12 hours. C-D The gMFIs of the indicated surface markers on heart ILC2s for both male and female mice after stimulation with the indicated concentrations of 17β-E2 (C) and testosterone (D) for 12 hours. Each dot represents one mouse; different colors represent different litters; error bars represent the mean ± SD; two-way ANOVA followed by Dunnett’s test (A-D). **Figure S3**: scRNA-Seq analysis of heart lymphocytes from both male and female mice related to Fig. 6. A UMAP reduction and data visualization of major heart CD45^+^ cells with high expression of CD45. After unsupervised clustering, different types of lymphocytes were identified by corresponding markers. In the total heart, ILC2s are highlighted by red circles. B Violin plots showing the expression of ILC2 marker genes (Gata3, Il7r) in heart CD45^+^ cells. C UMAP plot showing the heart lymphocyte cell types in male and female mice. **Figure S4**: Proportion of ILC2s in mouse heart tissues before or after FASC sorting. Evaluation of the purity of sorted heart ILC2s from one representative samples. Gating strategy of heart ILC2s sorting and the percentage of each gate are shown (Top: Pre-sort; Bottom: Post-sort). The purity of heart ILC2s after FASC sorting was 90.6%.

**Additional file 2.** DEGs of ILC2 and its clusters between female and male mice.

## Data Availability

All data are included in the manuscript. The datasets analyzed in the current study are available from the corresponding author on reasonable request.

## Abbreviations

ILC2s: Group 2 innate lymphoid cells
ILCs: Innate lymphoid cells
WT: Wild-type
Klrg1: Killer-cell lectin-like receptor G1
Sca-1: Stem cell antigen 1
IL: Interleukin
gMFI: Geometric mean fluorescence intensity
Ar: Androgen receptor
Er: Estrogen receptor
17β-E2: 17β-Estradiol
Xist: X-inactive specific transcript
scRNA-seq: Single-cell RNA sequencing
UMAP: Uniform manifold approximation and projection
DEGs: Differentially expressed genes
GSVA: Gene set variation analysis
Ddx3y: DEAD-box helicase 3 Y-linked
Klrk1: Killer cell lectin like receptor K
Il18r1: Il18 receptor 1
Fxr1: Fragile X mental retardation autosomal homolog 1
Arl5b: ADP ribosylation factor like GTPase 5b
Sema4a: Semaphorin 4a
Txnip: Thioredoxin interacting protein
Hilpda: Hypoxia-inducible lipid droplet-associated
Ccl3: C–C motif chemokine ligand 3
Pparg: Peroxisome proliferator activated receptor gamma
