# Supplementary material for: Sex differences exist in adult heart group 2 innate lymphoid cells
Source: BMC Immunol. 2022 Oct 31;23:52. doi: 10.1186/s12865-022-00525-0 (PMC9620621; doi:10.1186/s12865-022-00525-0)
Supplement: Supplementary file 1 — Additional file 1. Figure S1: The percentage and numbers of heart Gata3+ ILC2s in 8-week-old wild-type C57BL/6 mice. A Cumulative frequencies and enumeration of heart Gata3+ ILC2s among ILC2s (identified as CD45+Lin-CD127+CD90.2+ST2+ cells, left) or CD45+ lymphocytes (middle) in 8-week-old wild-type mice by flow cytometric analysis. B Cumulative frequencies and enumeration of heart GATA3+CD127+ ILC2s among CD45+ lymphocyte in 8-week-old wild type mice by flow cytometric analysis. Each dot represents one mouse; different colors represent different litters; error bars represent the mean ± SD; *p < 0.05, **p < 0.01. Unpaired two-tailed Student’s t test (A-B). Figure S2: The responsiveness of heart ILC2s to sex hormones. A-B The cumulative frequencies of heart ILC2s among CD45+ cells treated with the indicated concentrations of 17β-E2 (A) and testosterone (B) for 12 hours. C-D The gMFIs of the indicated surface markers on heart ILC2s for both male and female mice after stimulation with the indicated concentrations of 17β-E2 (C) and testosterone (D) for 12 hours. Each dot represents one mouse; different colors represent different litters; error bars represent the mean ± SD; two-way ANOVA followed by Dunnett’s test (A-D). Figure S3: scRNA-Seq analysis of heart lymphocytes from both male and female mice related to Fig. 6. A UMAP reduction and data visualization of major heart CD45+ cells with high expression of CD45. After unsupervised clustering, different types of lymphocytes were identified by corresponding markers. In the total heart, ILC2s are highlighted by red circles. B Violin plots showing the expression of ILC2 marker genes (Gata3, Il7r) in heart CD45+ cells. C UMAP plot showing the heart lymphocyte cell types in male and female mice. Figure S4: Proportion of ILC2s in mouse heart tissues before or after FASC sorting. Evaluation of the purity of sorted heart ILC2s from one representative samples. Gating strategy of heart ILC2s sorting and the percentage of each ga [file 12865_2022_525_MOESM1_ESM.docx]

**Sex differences exist in adult heart group 2 innate lymphoid cells**

Hongyan Peng ^1,2, *^, Shuting Wu ^1,2, *^, Shanshan Wang^3^, Qinglan Yang ^1,2^, Lili Wang ^1,2^, Shuju Zhang ^1,2^, Minghui Huang ^1,2^, Yana Li ^1,2^, Peiwen Xiong ^1,2^, Zhaohui Zhang ^4^, Yue Cai ^5^, Liping Li ^1,2^, Youcai Deng ^4, #^, Yafei Deng ^1,2, #^

**Supplementary Materials**

**Supplemental figures and figure legends**

^
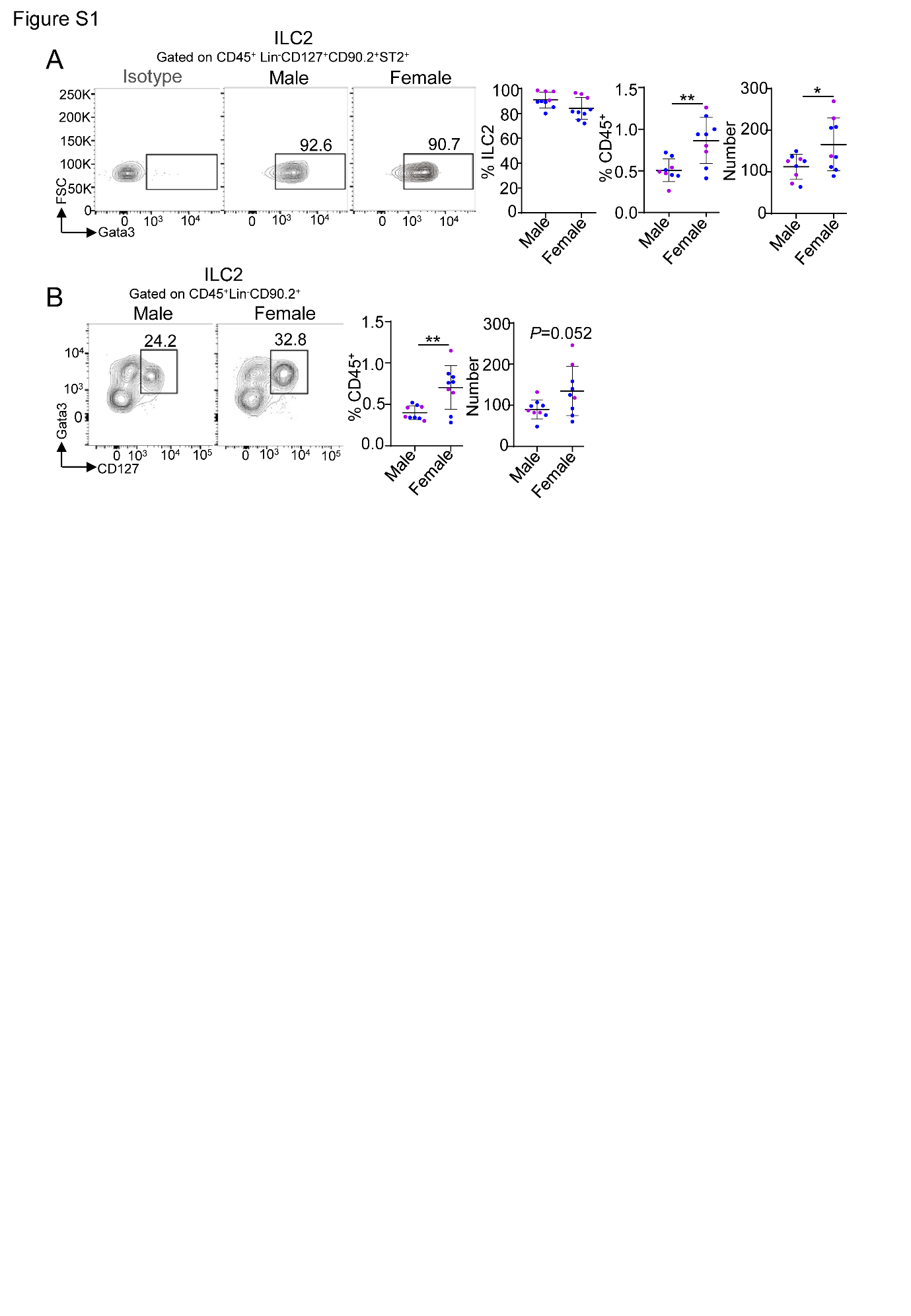
^

**Figure S1** The percentage and numbers of heart Gata3^+^ ILC2s in 8-week-old wild-type C57/B6 L mice. **A** Cumulative frequencies and enumeration of heart Gata3^+^ ILC2s among ILC2s (identified as CD45^+^ Lin^-^CD127^+^CD90.2^+^ST2^+^ cells, left) or CD45^+^ lymphocytes (middle) in 8-week-old wild-type mice by flow cytometric analysis. **B** Cumulative frequencies and enumeration of heart GATA3^+^CD127^+^ ILC2s among CD45^+^ lymphocyte in 8-week-old wild type mice by flow cytometric analysis. Each dot represents one mouse; different colors represent different litters; error bars represent the mean ± SD; **p* < 0.05, ***p* < 0.01. Unpaired two-tailed Student’s t test (A-B).


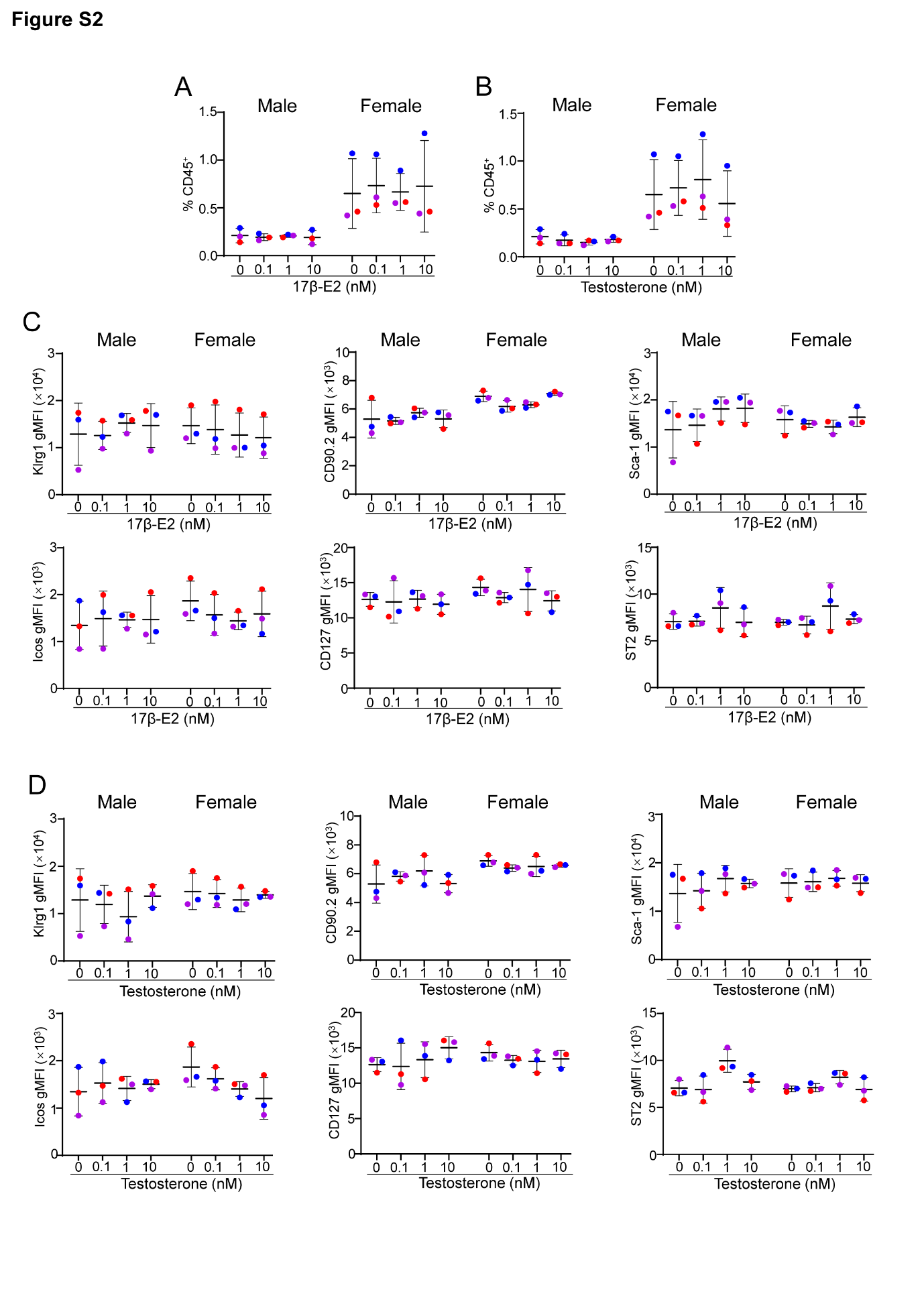


**Figure S2** The responsiveness of heart ILC2s to sex hormones. **A-B** The cumulative frequencies of heart ILC2s among CD45^+^ cells treated with the indicated concentrations of 17β-E2 (**A**) and testosterone (**B**) for 12 hours. **C-D** The gMFIs of the indicated surface markers on heart ILC2s for both male and female mice after stimulation with the indicated concentrations of 17β-E2 (**C**) and testosterone (**D**) for 12 hours. Each dot represents one mouse; different colors represent different litters; error bars represent the mean ± SD; two-way ANOVA followed by Dunnett’s test (A-D).


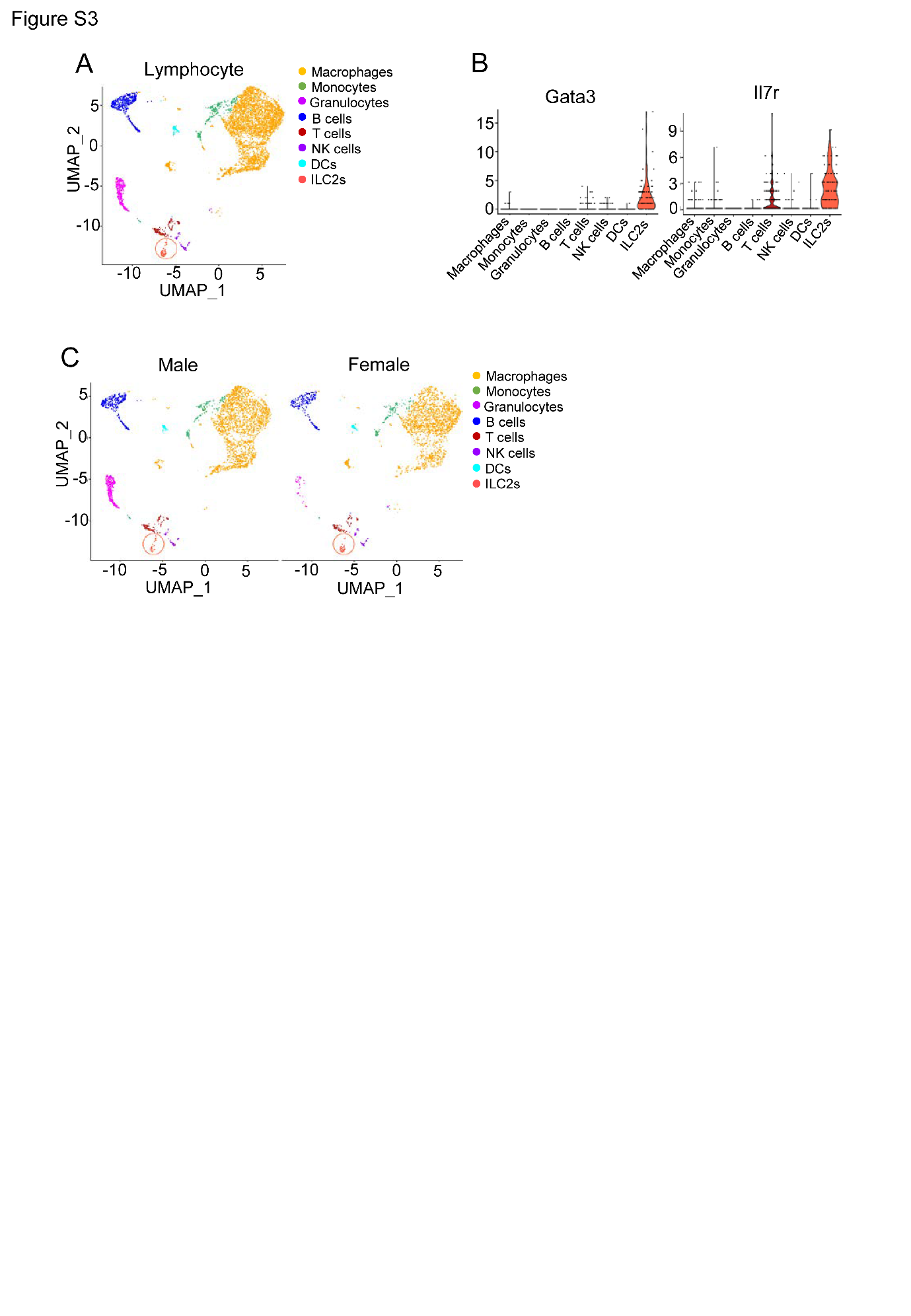


**Figure S3** scRNA-Seq analysis of heart lymphocytes from both male and female mice related to Fig. 6. **A** UMAP reduction and data visualization of major heart CD45^+^ cells with high expression of CD45. After unsupervised clustering, different types of lymphocytes were identified by corresponding markers. In the total heart, ILC2s are highlighted by red circles. **B** Violin plots showing the expression of ILC2 marker genes (*Gata3, Il7r*) in heart CD45^+^ cells. **C** UMAP plot showing the heart lymphocyte cell types in male and female mice.


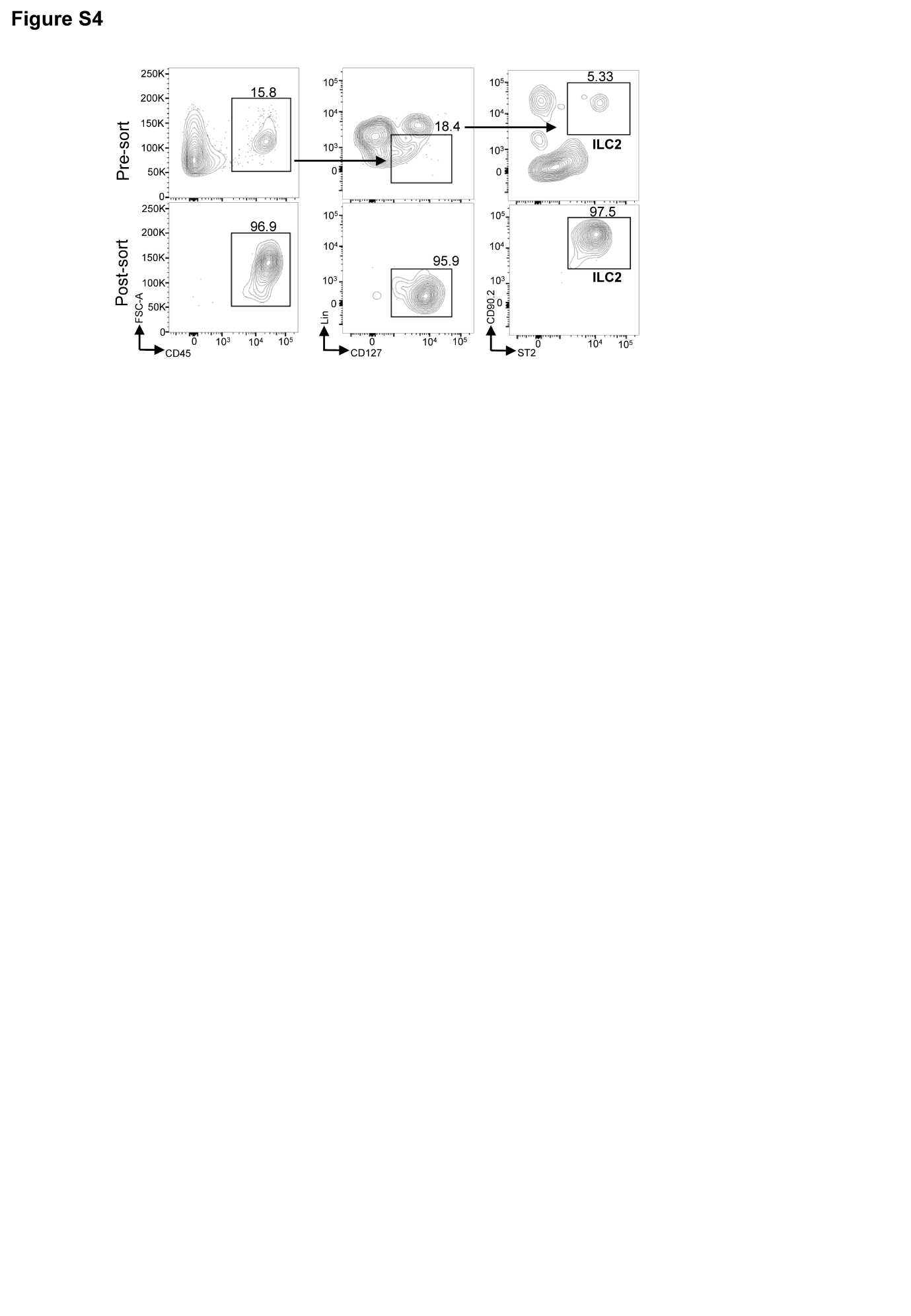


**Figure S4** Proportion of ILC2s in mouse heart tissues before or after FASC sorting. Evaluation of the purity of sorted heart ILC2s from one representative samples. Gating strategy of heart ILC2s sorting and the percentage of each gate are shown (Top: Pre-sort; Bottom: Post-sort). The purity of heart ILC2s after FASC sorting was 90.6%.
